# The health and economic impact of acute gastroenteritis in Belgium, 2010–2014

**DOI:** 10.1017/S095026881900044X

**Published:** 2019-03-12

**Authors:** Theofilos Papadopoulos, Sofieke Klamer, Stephanie Jacquinet, Boudewijn Catry, Amber Litzroth, Laure Mortgat, Pavlos Mamouris, Javiera Rebolledo, Bert Vaes, Dieter Van Cauteren, Johan Van der Heyden, Philippe Beutels, Brecht Devleesschauwer

**Affiliations:** 1 Department of Epidemiology and Public Health, Sciensano, Brussels, Belgium; 2 European Programme for Intervention Epidemiology Training (EPIET), European Centre for Disease Prevention and Control, (ECDC), Stockholm, Sweden; 3 Faculty of Medicine, Université Libre de Bruxelles (ULB), Brussels, Belgium; 4 Department of Public Health and Primary Care, Katholieke Universiteit Leuven (KU Leuven), Leuven, Belgium; 5 Centre for Health Economics Research and Modelling Infectious Diseases, Vaccine and Infectious Disease Institute, University of Antwerp, Antwerp, Belgium; 6 Department of Veterinary Public Health and Food Safety, Faculty of Veterinary Medicine, Ghent University, Merelbeke, Belgium

**Keywords:** Burden of disease, cost-of-illness, disability-adjusted life years, gastroenteritis

## Abstract

Acute gastroenteritis (AGE) remains a common condition in both low- and high-income countries. In Belgium, however, there is currently a lack of information on the societal health and economic impact of AGE. We conducted a retrospective study using mortality and cause-of-death data, hospital data, primary care data, health interview survey data and other published data. We estimated the burden of illness during a 5-year period (2010–2014) in Belgium in terms of deaths, patients admitted to hospitals, patients consulting their general practitioner (GP) and cases occurring in the community. We further quantified the health impact in terms of disability-adjusted life years (DALYs) and the economic impact in terms of cost-of-illness estimates. We estimated 343 deaths, 27 707 hospitalised patients, 464 222 GP consultations and 10 058 741 episodes occurring in the community (0.91 cases/person) on average per year. AGE was associated with 11 855 DALYs per year (107 DALY per 100 000 persons). The economic burden was estimated to represent direct costs of €112 million, indirect costs of €927 million (90% of the total costs) and an average total cost of €103 per case and €94 per person. AGE results in a substantial health and economic impact in Belgium, justifying continued mitigation efforts.

## Introduction

Acute gastroenteritis (AGE) is a common condition causing a significant disease burden worldwide. The Global Burden of Disease study estimated that diarrhoea was a leading cause of death with 1.7 million deaths in 2016 [1]. Although in high-income countries mortality due to diarrhoeal diseases is low, AGE gives rise to numerous episodes, general practitioner (GP) consultations and hospitalisations. The number of deaths, hospitalised patients, GP consultations and cases occurring in the community can be described in a disease pyramid, allowing for a more complete picture of the burden of illness [2, 3]. The disease pyramid further provides a basis for quantifying the health and economic impact of AGE [4, 5].

Different data sources provide information on the AGE disease pyramid in Belgium, albeit with varying degrees of completeness. All deaths and hospitalisations are registered and classified according to ICD coding. Systematic collection of data on AGE from GPs is however more limited. Furthermore, AGE cases occurring in the community are difficult to estimate as not all cases seek health care, especially when the disease is mild and self-limiting.

The aim of this study was to assess the health and economic impact of AGE in Belgium using available data sources.

## Materials and methods

### Reference period and population

We performed a retrospective analysis of routinely collected health information from different sources targeting the Belgian population for the years 2010–2014. This time period provided the most complete coverage across databases. First, we reconstructed the AGE disease pyramid by estimating the number of episodes, GP consultations, hospitalisations and deaths occurring annually in Belgium. Then, we quantified the health impact in terms of disability-adjusted life years (DALYs) and the economic impact in terms of direct and indirect costs.

### Reconstruction of the disease pyramid using different data sources

**Fig. 1.**
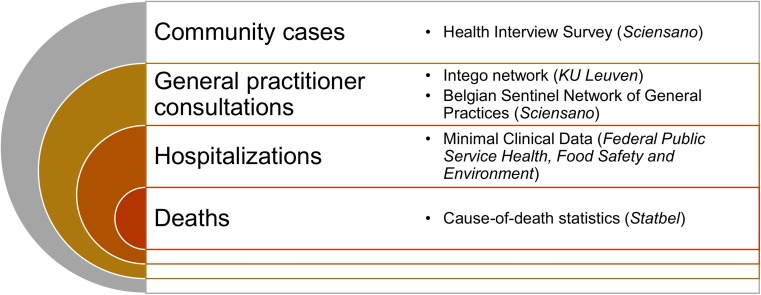
Data sources used to reconstruct the acute gastroenteritis disease pyramid in Belgium.

Figure 1 summarises the different steps in the AGE disease pyramid and the data sources mapped to each step. In what follows, we describe the mapping for each step in more detail.

## Mortality and cause-of-death data

Statbel, the Belgian national institute of statistics, is responsible for the compilation of mortality and ICD-10 coded cause-of-death data based on death certificates. In the current study, only the underlying cause of death was taken into account. The underlying cause of death is defined in accordance with the encoding rules of the World Health Organization (WHO), as the diseases or the injuries which initiate a chain reaction of morbidities that finally led to death [6]. Cause-of-death data can be extracted through the Standardized Procedures for Mortality Analysis website (SPMA), available online through https://spma.wiv-isp.be/. We extracted data for 2010–2014 from SPMA to establish the number of deaths with the ICD-10 codes A00-09 (‘Diarrhoea and gastroenteritis of presumed infectious origin’) as the underlying cause of death.

## Hospital data

The Minimal Clinical Data (MCD) is a compulsory hospital data registration system, providing summarised clinical and demographic information for all persons admitted to Belgian hospitals. Participation to the MCD has been mandatory for all hospitals since 1991, and is managed by the Federal Public Service Health, Food Safety and Environment. Hospital data are available for hospitalised episodes of AGE as a primary (main) or secondary (any non-primary) diagnosis, coded by ICD-9 until 2014. In 2015, the database shifted to ICD-10 coding, but data were not available for the year 2015. We obtained the annual number of hospitalised AGE cases during 2010–2014, by age, from MCD as the number of persons admitted to hospital with AGE defined by ICD-9 codes 001-009 as a primary or secondary diagnosis during their hospital stay.

## Primary care data

The two major sources of primary care data in Belgium are the Belgian Sentinel Network of General Practices (SNGP) [7], managed by Sciensano, the Belgian institute for health, and the Intego network, managed by the Katholieke Universiteit Leuven (KU Leuven) [8].

The Belgian SNGP comprises approximately 150 general practices with one or more sentinel GPs who purposively record routine clinical care data for the surveillance of different health problems. The network covers between 1.4% and 1.8% of the Belgian population throughout all regions. AGE was included in the SNGP network surveillance in 2002. For each case, a questionnaire was completed containing patient characteristics and specific questions about the symptoms (number of loose stools/day, fever, blood in stool, vomiting and dehydration), antibiotic prescription and if a stool sample was requested. Information on the age and gender distribution of the sentinel population is lacking, therefore the same age and gender distribution as in the complete Belgian population is assumed when calculating the incidence rates. For the SNGP, a case of AGE was defined as any episode with at least four loose stools/day or loose stool/vomiting in combination of at least two other symptoms (fever or blood in stool).

The Intego network, operational since 1994, is an electronic patient record (EPR)-based network of 54 voluntarily participating GP practices in Flanders, the northern region of the country, which all use the same EPR software. The network is coordinated by the Academic Centre for General Practice at the KU Leuven and covers approximately 2% of the Flemish population. The Intego database primarily uses the International Classification of Primary Care (ICPC) coding system to register GP diagnoses. In this coding system, AGE may be classified as ICPC code D70 (‘gastrointestinal infection’) or D73 (‘gastroenteritis presumed infection’).

To estimate the number of GP consultations for AGE, we first extracted from the Intego database the number of GP registrations in Flanders with ICPC codes D70 or D73, by age, for the period 2010–2014. We then used the SNGP data for 2002 to extrapolate the number of consultations to the other regions (i.e. the Walloon and Brussels Capital Region), based on the proportion of AGE-related consultations per region in 2002.

## Health interview survey data

The Belgian Health Interview Survey (BHIS) has, to date, taken place in 1997, 2001, 2004, 2008 and 2013 [9]. The main objective of the BHIS is to describe in a representative manner the health status, health behaviour and use of health services of the Belgian population. The sampling design is a stratified clustered multi-stage with municipalities as primary, households as secondary and individuals as the tertiary sampling units. Questions on AGE have only been included in the BHIS 2001. In 2001, a total of 12 111 people were interviewed. The AGE-specific questions asked if the participant had had an episode of AGE in the last 2 weeks prior to interview. Cases of AGE in the BHIS 2001 were defined as every person who reported three or more loose stools in a 24 h period during the 14 days prior to the interview, without having reported having chronic bowel problems during the past 6 months.

The number of community cases was estimated from the BHIS 2001 considering an average duration of symptoms of 1–5 days [10]. In a baseline scenario, we estimated the number of community cases by multiplying the number of positive respondents with 365/17, considering an average duration of symptoms of 4 days (17 = 14 days + [4–1] days of symptoms). In alternative incidence scenarios, we estimated the minimum number of cases considering a 5-day duration of symptoms (number of positive responses × 365/18), and the maximum number assuming the minimum duration of 1 day for all cases (number of positive responses × 365/14). In 2006, a rotavirus vaccine came on the market in Belgium, and quickly achieved high uptake (mean uptake in 2012: 89%) [11]. We therefore further corrected the community incidence for children 0–9 years old by applying the post-/pre-vaccination proportionate decrease (76.9%) in AGE hospitalisations for this age group, as estimated previously [12] and assuming that the decrease in community incidence would be the same as in hospitalisations.

### Disability-adjusted life years

The burden of AGE was evaluated in terms of DALYs, a summary measure of population health that is widely used to quantify burden of disease. DALYs were calculated as previously described [13] by summing years of life lost (YLLs) and years lived with disability (YLDs). Estimations for YLLs were performed using the standard life expectancy table from the Global Burden of Disease study [1]. For the estimations of YLDs, we stratified AGE cases into mild, moderate and severe and matched these strata with the levels across the disease pyramid (i.e. cases occurring in the community, GP visits and hospitalisations, respectively). To ensure that each case would be categorised in one part of the disease pyramid only we excluded the number of severe cases from the moderate cases and the number of moderate and severe cases from the number of mild cases. In a baseline scenario, we used the disability weights from Salomon *et al*. [14] – i.e. 0.074, 0.188 and 0.247, for mild, moderate and severe AGE, respectively. The duration of symptoms was set as 3 days for mild AGE (community cases), 10 days for moderate AGE (when a GP was consulted) and 14 days for severe AGE (hospitalisation cases) [15]. In an alternative disability weight scenario, we used the annual profile disability weights from Haagsma *et al*. [15] – i.e. 0.000, 0.015 and 0.041 for mild, moderate and severe AGE. DALYs are presented as the total number in Belgium per year, as number of DALYs per 100 000 persons and as number of DALYs per 100 000 cases.

### Cost-of-illness

The economic impact of AGE was calculated considering direct medical costs, direct non-medical costs and indirect costs. We estimated the total cost-of-illness from a societal perspective using data on the total number of AGE cases per age group, the volumes (number of consultations, hospitalisations, medication packages) for use of resources and the unit costs of each of these items. As such, the average costs per case in a certain age group was calculated and subsequently multiplied by the total number of cases in each age group [5, 16, 17]. Then, the total absolute cost-of-illness of AGE in Belgium and per case was calculated by summing costs across all age groups. Our cost-of-illness estimates were in line with the Belgian guidelines for economic evaluations and budget impact analyses, developed by the Belgian Healthcare Knowledge Centre (KCE) [18].

## Direct costs

We estimated the total direct medical costs as the sum of the costs for consultations at GPs and/or specialists, hospital admissions, prescribed and over-the-counter medication, and laboratory stool tests for diagnostic purposes. Unit costs for GP or specialist consultations as well as for hospitalisations were obtained from the National Institute of Health and Disability Insurance (NIHDI) for 2016 (www.inami.fgov.be). The direct cost per GP consultation was €24.48. For consultation of gastroenterologist, a cost of €36.74 was used, and in the absence of reliable data, we assumed that a 14% fraction of the people that visited a GP consulted a specialist [19]. We used a mean duration of 4.4 days per hospitalisation and a mean cost of €2800 per hospitalisation day as estimated by NIHDI (https://tct.fgov.be/webetct/etct-web/). For the prescribed and the over-the-counter medications, we considered three main categories: antidiarrhoeal drugs (AD, domperidone; €7.2 per package); oral rehydration solutions (ORS; €9 per package); and antibiotics (AB, amoxicillin and clavulanic acid; €10.41 per package) (prices derived from the Belgian Center for Pharmacotherapeutic Information). The proportions of episodes in which these drugs were used, i.e. 4.3% for AD, 7.4% for ORS and 6.5% for AB, were taken from a previous study [5]. For the diagnostic stool tests, we assumed that 13.5% of the GP visits resulted in the prescription of diagnostic stool tests with a unit cost of €60 per test [5].

For the direct non-medical costs, we considered travel costs to and from the GP and the hospital as previously estimated in the Netherlands [5]. The average distance from a Belgian household to their GP was set to be 1.8 km as in the Dutch study, considering the same population density. We further assumed that half of the patients used a car (or other paid transport), while the other half used a bicycle or went on foot [5]. The average distance from a Belgian household to the nearest general hospital was set to be 7.0 km as in the Dutch study; we further assumed that all patients used paid transport to go to the hospital [5]. For all paid transports, we used a cost of €0.30/ km [18] and no parking costs were included. We did not consider costs for additional cleaning material and diapers for patients in the absence of reliable data for the estimations. For the pharmacy, we did not estimate any additional costs as we considered that usually people went on foot or bought the medication on the way to the GP.

## Indirect costs

We associated indirect costs with losses due to absence from work using two scenarios. According to the baseline scenario, we assumed that in 37% of adult episodes (18–64), and in 18.6% of the episodes in children (0–17) and seniors (65+), an employed adult had to be absent from work due to AGE, as previously described in a recent survey in the Netherlands [20]. The average number of working days that an employee was absent from work due to gastroenteritis (3.5 days) was retrieved from Securex, one of the largest human resource companies in Belgium. The number of absenteeism episodes was multiplied with the average number of absence days from work per episode and then multiplied with the average daily gross salary of €257 (2010 average) [18] per working day to estimate the total costs.

According to an alternative scenario for estimating indirect costs, we assumed that only people who asked for medical advice, i.e. the moderate or severe cases, were absent from work. Indeed, in Belgium, a medical certificate is needed to justify absence from work. We estimated the workdays lost by multiplying the sum of people that visited a GP, a specialist or had been hospitalised with the average number of working days that an employee was absent from work due to gastroenteritis (3.5 days) as described above. We considered age group-specific employment rates using Eurostat data for 2012 – i.e. 25.3% for ages 18–24, 79.3% for ages 25–54 and 39.5% for ages 55–64. We assumed that for children (0–17) and elderly people (65+), at least one productive adult was taking care of them and was absent from work for 1 day in case of a moderate episode and for 2 days in case of a severe episode [17].

## Results

### Disease pyramid

The disease pyramid of AGE in Belgium is presented in Table 1. We estimated an average of 10 058 741 community cases per year in Belgium during the study period, corresponding to 0.91 cases/person per year (range 0.86–1.1 cases/person per year) in an average population of 11 052 385. The alternative incidence scenarios yielded a minimum and a maximum estimate of 9.5 and 12.2 million community cases, respectively. Cause-of-death data yielded an average of 343 (217–408) AGE deaths per year, corresponding to 3.1 deaths/100 000 persons, or 2.9 deaths/100 000 community cases (Supplementary Table S1). Hospital data yielded an average of 27 707 (26 312–29 217) AGE hospitalisations per year, corresponding to 251 hospitalisations/100 000 persons or 275 hospitalisations/100 000 community cases. Based on primary care data, we estimated 464 222 GP consultations due to AGE on average per year (460 187–466 602), corresponding to 4200 consultations/100 000 persons, or 4450 consultations/100 000 community cases.

**Table 1.** Estimated disease pyramid of acute gastroenteritis cases in Belgium, 2010–2014

Component	Number of cases					
	*2010*	*2011*	*2012*	*2013*	*2014*	*Average*
Deaths	408	362	217	387	339	343
Hospitalisations	27 699	26 652	28 654	29 217	26 312	27 707
GP consultations	460 187	463 267	465 081	465 975	466 602	464 222
Community cases, baseline estimate	9 920 001	10 010 579	10 075 302	10 121 939	10 165 885	10 058 741
Community cases, minimum estimate	9 368 890	9 454 436	9 515 563	9 559 610	9 601 114	9 499 922
Community cases, maximum estimate	12 045 716	12 155 704	12 234 296	12 290 926	12 344 289	12 214 186

GP, general practitioner.

### Disability-adjusted life years

During 2010–2014, the fatal AGE cases resulted in an annual average of 3509 YLLs. This corresponds to an annual rate of 32 YLLs per 100 000 persons and 35 YLLs per 100 000 cases (Table 2). In our baseline disability weights scenario, 8346 years of life were lost on average per year due to disability in Belgium, corresponding to 83 YLDs per 100 000 cases/year and 76 YLDs per 100 000 persons/year. Mild AGE cases contributed the most to the YLD estimate (70%), followed by moderate (27%) and severe cases (3%) (Table 2). In total, AGE was responsible for 11 855 DALYs per year in Belgium during 2010–2014, corresponding to 107 DALYs per 100 000 persons and 118 DALYs per 100 000 cases.

**Table 2.** Estimated annual disease burden of acute gastroenteritis (AGE) in Belgium, 2010–2014 (baseline scenario)

Health state	Cases	YLDs	YLLs	DALYs	DALYs/1000 cases^a^	DALYs/100 000 total cases	DALYs/100 000 persons
AGE, mild	9 594 519	5836	N/A	5836	0.61	58	53
AGE, moderate	436 515	2248	N/A	2248	5.15	22	20
AGE, severe	27 707	262	N/A	262	9.47	2.61	2.37
AGE, deaths	343	N/A	3509	3509	10 229	35	32
TOTAL	N/A	8346	3509	11 855	N/A	118	107

a DALYs per 1000 mild, moderate or severe cases, respectively.

YLDs, years lived with disability; YLLs, years of life lost; DALYs, disability-adjusted life years; N/A, not applicable.

Using the alternative disability weights scenario, we estimated 6548 YLDs for the moderate cases and 1136 YLDs for the severe cases of AGE, while this approach resulted in zero YLDs for mild cases. Consequently, the total estimated DALYs were 11 192, corresponding to 101 DALYs per 100 000 persons and 111 DALYs per 100 000 cases (Supplementary Table S2).

### Cost-of-illness

We estimated the direct medical costs at €112 million, or €11 per case and €10 per person, accounting for 11% of the total costs (Table 3). Nearly 70% of direct medical costs were due to hospitalisation (€78 million), while another 15% was due to the medication (€17 million). The direct non-medical costs (i.e. the patients’ transport costs) were lower, contributing only €402 144. We estimated the indirect costs as €927 million accounting for 90% of the total costs according to baseline scenario, or €85 per case and €84 per person. In the alternative indirect cost scenario, we estimated an indirect cost of €98 million or 47% of total costs, corresponding to €10 per case and €9 per person (Table 3).

**Table 3.** Estimated annual direct and indirect costs of acute gastroenteritis in Belgium, 2010–2014

	Unit cost^a^	Average estimated units used per year	Total costs
TOTAL COSTS			1 039 451 369
Direct costs			112 167 401
*Direct medical costs*			*111 765 256*
Consultation with a general practitioner	24.48	464 222	11 364 155
Consultation with a specialist	36.74	64 991	2 387 772
Hospitalisation	2800	27 707	77 579 600
Antibiotics	10.41	653 974	6 807 868
Oral rehydration solutions	9	747 399	6 726 588
Antidiarrhoeals	7.2	435 983	3 139 075
Stool tests	60	62 670	3 760 198
*Direct non-medical costs*			*402 144*
Transport from and to the doctor	1.08	264 607	285 775
Transport from and to the hospital	4.2	27 707	116 369
Indirect costs			*927 283 968*
Productivity losses – children	257	272 795	70 108 224
Productivity losses – adults	257	3 335 314	857 175 744

a Prices in euros.

In Belgium, the annual total costs for AGE under the baseline scenario amounted to €1 billion on average during the study period. This is equivalent to an average of €103 per case and €94 per person (Table 3). Using the alternative indirect cost scenario, we estimated the total costs at €210 million, or €21 per case and €19 per person (Table 4).

**Table 4.** Estimated annual indirect and total costs of acute gastroenteritis in Belgium, 2010–2014 (alternative scenario)

	Unit cost^a^	Average estimated units used per year	Total costs
TOTAL COSTS			210 060 243
Indirect costs			*97 892 842*
Productivity losses 0–17	257	100 528	25 835 696
Productivity losses 18–24	257	16 714	4 295 498
Productivity losses 25–54	257	197 472	50 750 304
Productivity losses 55–64	257	13 510	3 472 070
Productivity losses 65+	257	52 682	13 539 274

a Prices in euros.

## Discussion

We performed to our knowledge the first assessment of the health and economic burden of all-cause AGE in Belgium. During 2010–2014, we estimated 0.91 AGE cases per person per year, resulting in 12 000 DALYs and a total cost of €1 billion, corresponding to €103 per AGE case when productivity losses were included. The estimated costs when productivity losses related to time off from work were excluded were considerably less, namely €112 million at an average cost per case of €11.

As in other high-income countries, AGE is common and represents a significant burden to society in Belgium. Our AGE incidence (0.91 cases/person per year) is at the lower end of the range of AGE incidences estimated from retrospective surveys in other high-income countries [21–24]. A review of estimates of the incidence and prevalence of AGE from 33 studies from high-income countries has shown a range from 0.1 to 3.5 episodes per person year [25]. However, our estimates for the episodes occurring in the community are based on the BHIS conducted nearly 20 years ago and it is unknown if the incidence has changed through these years. Although our estimates were similar to most other countries, more recent data are necessary to confirm the current burden of AGE at community level.

Interestingly, the implied proportion of cases consulting a GP (5%) was at the lower end of the range of reported estimates from other countries, i.e. 4–33% [5, 21, 26, 27]. Nevertheless, the GP consulting rate was expected to be high in Belgium as a GP certificate is generally needed to justify absence from work. These discrepancies might imply an overestimation of the number of community cases in our study, or an underestimation of the number of GP consultations, or both. The discrepancies could also be the result of differences in study design or case definitions across studies, or of different healthcare systems and healthcare-seeking behaviour across countries.

DALY calculations complement information on disease incidence and mortality. Our estimate of 118 DALYs per 100 000 cases, which represents the patient burden, is very low in comparison with most of the infectious diseases occurring in several countries in EU and globally [28–30]. However, the total burden of the disease per 100 000 persons, driven by the large number of episodes, is higher than each of the 31 communicable diseases in a recent European study, including influenza, tuberculosis and HIV/AIDS [28]. Our results further indicated that the DALYs associated with mortality were only a small fraction of the total DALYs (30%), while the morbidity impact accounted for 70%. This is a reflection of the low case fatality ratio of AGE in high-income countries, particularly compared with low-income countries, where mortality remains the dominant contributor to the AGE disease burden [30].

Studies on the economic burden of AGE conducted in other countries mainly focused on only health care costs [31, 32], gastrointestinal disease (acute and chronic) [33–35], foodborne gastroenteritis [36–38] or specific pathogens [31, 39, 40]. Therefore, their results are difficult to compare with ours. The estimated economic burden (including direct and indirect costs) of gastrointestinal infections or foodborne illnesses in high-income countries varies between €14 in Australia and €1305 in the USA per case [31]. The estimates of a study conducted in the Netherlands in 2004 yielded estimates comparable with ours – i.e. a total cost of €77 per case and indirect costs representing 82% of the total costs [5].

Our study has several limitations. First and foremost, our disease pyramid estimates are limited by the inherent limitations of each of the applied data sources. For the community cases, the only available database was the BHIS performed in 2001. Since this database has a very short recall period (14 days) and probably overestimates the real incidence of the disease [26], we corrected the estimates by inserting the disease duration in the calculations. Our scenario analyses based on a minimum and a maximum duration of symptoms of 0 and 5 days showed that our estimate was relatively robust against this assumption. For the GP consultations, the only available nationally representative data (SNGP) were for the year 2002, while the most recent data (Intego) were only available for Flanders. Furthermore, it is possible that one AGE event resulted in more than one GP consultation; however, Intego data show that for the vast majority of events, only one consultation was registered. Finally, our death estimates were derived from cause-of-death data considering only the underlying cause. Nonetheless, AGE could have contributed to the death without being listed as the underlying cause. Over the 5-year study period, in addition to the 1713 AGE deaths, another 297 (59 per year) had ICD-10 codes A00-09 listed in the chain of events leading to death, but without being the underlying cause of death. Including these deaths would have resulted in a 17% increase in the number of AGE deaths, and a consequent increase in the number of YLLs and DALYs. However, it is common practice in burden of disease studies to assign deaths only to the underlying cause of death, thus avoiding double-counting when deaths would be assigned to more than one cause.

In addition to the inherent biases in the individual data sources, across data sources, case definitions were not constant. The exact impact of this limitation is difficult to ascertain. Furthermore, we used a 5-year reference period, 2010–2014, to average out possible aberrant temporal variations. The study period was further driven by the availability of data across data sources. In particular, the last available MCD data according to ICD-9 classification were from 2014; in 2015, MCD data were not available due to transforming to ICD-10 coding.

For the DALY calculations, we used two different approaches for defining morbidity-associated health losses, in line with current major studies [14, 40]. Interestingly these two estimations were only 9% different, suggesting that the overall estimates are robust. However, the YLD estimates by severity level did differ significantly.

Our cost-of-illness estimates were generally limited by a lack of recent and nationally representative data on healthcare use and productivity losses, and should therefore be interpreted with caution. The current estimates are therefore partially based on data from other countries [5], data from studies regarding different diseases [17, 19] or expert advices. Data were particularly lacking for estimating indirect costs. We considered two scenarios for estimating the extent of absenteeism among patients and caregivers, which resulted in widely different estimates, ranging from €927 million in our baseline scenario to €98 million in a more conservative alternative scenario. The important contribution of productivity losses clearly warrants further nationally representative surveys to address this gap. A key data gap was the absenteeism associated with AGE in older adults (65+), who account for an important proportion of the AGE cases. As most surveys focus on AGE in children and their parents, little is known about the consequences for older adults and their caregivers. In both scenarios, we therefore assumed the same levels of absenteeism for caregivers of children and elderly adults, which may have been an overestimation of the indirect cost. Furthermore, we excluded certain cost components due to general lack of data, such as non-specified over-the-counter medication, taxi use, parking expenses or additional cleaning material and diapers. We also did not consider the possible use of leftovers from previously bought medication from the patients with AGE, which led to an overestimation in our cost estimates; however, the effect of these aspects on the total cost estimates are not likely to be substantial.

Despite these limitations, our results indicate major health and economic losses associated with AGE morbidity in Belgium. To support risk management, further studies are needed to unravel the relative contribution of specific pathogens to the AGE burden, as well as the role of community-acquired *vs.* hospital-acquired AGE.

## Conclusions

AGE results in a substantial health and economic impact in Belgium, justifying continued mitigation efforts. Nationally representative surveys are needed for addressing several of the identified data gaps, while further research is needed to identify the aetiologies underlying AGE in Belgium to support appropriate interventions.

## Supplementary material

For supplementary material accompanying this paper visit https://doi.org/10.1017/S095026881900044X. click here to view supplementary material
