# Supplementary material for: The health and economic impact of acute gastroenteritis in Belgium, 2010–2014
Source: Epidemiol Infect. 2019 Mar 12;147:e146. doi: 10.1017/S095026881900044X (PMC6518509; doi:10.1017/S095026881900044X)
Supplement: Supplementary file 1 [file S095026881900044Xsup001.zip › age-burden_tableS2.docx]

Epidemiology and Infection

The health and economic impact of acute gastroenteritis in Belgium, 2010–2014

Theofilos Papadopoulos, Sofieke Klamer, Stephanie Jacquinet, Boudewijn Catry, Amber Litzroth, Laure Mortgat, Pavlos Mamouris, Javiera Rebolledo, Bert Vaes, Dieter Van Cauteren, Johan Van der Heyden, Philippe Beutels, Brecht Devleesschauwer

**Supplementary Material**

## Supplementary Table S2: Estimated annual disease burden of acute gastroenteritis (AGE) in Belgium, 2010-2014 (alternative scenario).

| **Health state** | **Cases** | **YLDs** | **YLLs** | **DALYs** | **DALYs/**  **1,000 cases^a^** | **DALYs/**  **100,000 total cases** | **DALYs/**  **100,000**  **persons** |
| --- | --- | --- | --- | --- | --- | --- | --- |
| AGE, mild | 9,594,519 | 0 | N/A | 0 | 0 | 0 | 0 |
| AGE, moderate | 436,515 | 6,548 | N/A | 6,548 | 15 | 65 | 59 |
| AGE, severe | 27,707 | 1,136 | N/A | 1,136 | 41 | 11 | 10 |
| AGE, deaths | 343 | N/A | 3,646 | 3,646 | 10,229 | 35 | 32 |
| **TOTAL** | **N/A** | **7,684** | **3,509** | **11,192** | **N/A** | **111** | **101** |

^a^DALYs per 1,000 mild, moderate or severe cases, respectively

*YLDs: years lived with disability; YLLs: years of life lost; DALYs: disability-adjusted life years; N/A: not applicable*
