# Supplementary material for: The health and economic impact of acute gastroenteritis in Belgium, 2010–2014
Source: Epidemiol Infect. 2019 Mar 12;147:e146. doi: 10.1017/S095026881900044X (PMC6518509; doi:10.1017/S095026881900044X)
Supplement: Supplementary file 1 [file S095026881900044Xsup001.zip › age-burden_tableS1.docx]

Epidemiology and Infection

The health and economic impact of acute gastroenteritis in Belgium, 2010–2014

Theofilos Papadopoulos, Sofieke Klamer, Stephanie Jacquinet, Boudewijn Catry, Amber Litzroth, Laure Mortgat, Pavlos Mamouris, Javiera Rebolledo, Bert Vaes, Dieter Van Cauteren, Johan Van der Heyden, Philippe Beutels, Brecht Devleesschauwer

**Supplementary Material**

## Supplementary Table S1: Estimated annual deaths and incidence per 100,000 persons and 100,000 community cases due to acute gastroenteritis in Belgium, 2010-2014.

|  | **Deaths** | | | | | | **Average population 2010-2014** | **Incidence per 100,000 persons** | **Community cases**  **(baseline estimation)** | **Deaths per 100,000 community cases** |
| --- | --- | --- | --- | --- | --- | --- | --- | --- | --- | --- |
| **Age group** | **2010** | **2011** | **2012** | **2013** | **2014** | **Mean**  **2010-2014** |  |  |  |  |
| 0-4 | 0 | 1 | 0 | 2 | 0 | 0.6 | 643,802 | 0.09 | 73,450 | 0.82 |
| 5-9 | 0 | 0 | 0 | 1 | 1 | 0.4 | 621,066 | 0.06 | 38,552 | 1.04 |
| 10-14 | 0 | 0 | 0 | 0 | 0 | 0 | 610,603 | 0.00 | 565,448 | 0.00 |
| 15-19 | 0 | 0 | 0 | 0 | 0 | 0 | 636,017 | 0.00 | 520,414 | 0.00 |
| 20-24 | 0 | 0 | 0 | 0 | 0 | 0 | 687,927 | 0.00 | 830,061 | 0.00 |
| 25-29 | 0 | 0 | 1 | 1 | 0 | 0.4 | 698,068 | 0.06 | 928,106 | 0.04 |
| 30-34 | 0 | 0 | 0 | 0 | 1 | 0.2 | 723,907 | 0.03 | 1,040,657 | 0.02 |
| 35-39 | 1 | 2 | 2 | 0 | 0 | 1.0 | 727,188 | 0.14 | 804,975 | 0.12 |
| 40-44 | 0 | 2 | 0 | 1 | 2 | 1.0 | 780,516 | 0.13 | 764,215 | 0.13 |
| 45-49 | 1 | 2 | 0 | 2 | 2 | 1.4 | 817,285 | 0.17 | 790,817 | 0.18 |
| 50-54 | 5 | 4 | 0 | 5 | 2 | 3.2 | 793,792 | 0.40 | 669,631 | 0.48 |
| 55-59 | 5 | 4 | 2 | 2 | 4 | 3.4 | 720,557 | 0.47 | 562,574 | 0.60 |
| 60-64 | 11 | 6 | 4 | 5 | 5 | 6.2 | 650,523 | 0.95 | 715,116 | 0.87 |
| 65-69 | 11 | 11 | 8 | 13 | 18 | 12.2 | 533,084 | 2.29 | 461,517 | 2.64 |
| 70-74 | 28 | 18 | 10 | 21 | 14 | 18.2 | 433,547 | 4.20 | 352,595 | 5.16 |
| 75-79 | 48 | 40 | 29 | 38 | 36 | 38.2 | 397,958 | 9.60 | 252,448 | 15.13 |
| 80-84 | 81 | 71 | 35 | 75 | 80 | 68.4 | 313,317 | 21.83 | 397,511 | 17.21 |
| 85+ | 217 | 201 | 126 | 221 | 174 | 187.8 | 263,227 | 71.35 | 290,656 | 64.61 |
